# Implications of global health funding patterns for implementation of a novel soil-transmitted helminth and lymphatic filariasis medicine: Ivermectin/albendazole fixed-dose combination

**DOI:** 10.1371/journal.pntd.0014458

**Published:** 2026-07-09

**Authors:** Israel Wuresah, Alan Brooks, Nikita Kubal, Marina Gold, Alejandro Krolewiecki, Julie Jacobson

**Affiliations:** 1 Bridges to Development, Geneva, Switzerland; 2 Bridges to Development, Vashon, Washington, United States of America; 3 Mundo Sano Foundation, Madrid, Spain; 4 Instituto de Investigaciones de Enfermedades Tropicales/CONICET, Universidad Nacional de Salta, Orán, Argentina; University of Liverpool, UNITED KINGDOM OF GREAT BRITAIN AND NORTHERN IRELAND

## Abstract

**Background:**

Medicine donation programs have been central to neglected tropical disease (NTD) control, particularly for soil-transmitted helminths (STH), but growing concerns about sustainability, supply continuity, and country ownership highlight the need for new access models. A novel fixed-dose combination (FDC) of ivermectin and albendazole, recently endorsed by the European Medicines Agency, offers programmatic advantages over monotherapy. Unlike some existing treatments, the FDC may not be donated. This paper examines how the current drug donation landscape for STH shapes the prospects for future supply and financing of the FDC.

**Methods:**

Evidence for this paper was synthesized from a structured review of peer-reviewed and gray literature, supplemented by expert consultation. Data drawn from WHO’s Service Availability and Readiness Assessment (SARA) database on essential medicine availability, estimates of public health spending, and the World Bank’s World Development Indicators on health expenditure support the qualitative reports from sampled sources. Findings were interpreted within a framework examining three interrelated determinants of sustainable access: facility-level availability of essential medicines, health financing patterns, and existing donation models.

**Results:**

The analysis involved the review of 35 sources on essential medicine availability, financing, and supply of critical medical products, and medicine donation. SARA data showed striking gaps in essential medicine availability in health facilities, with some low- and lower-middle-income countries (LICs, LMICS) reporting zero facilities with sustainable availability. On health financing, African and Asian countries are spending only about half of what is called for in targets. Low- and lower-middle income countries remain heavily dependent on external aid and out-of-pocket spending, in stark contrast to high- and upper-middle and high-income countries where domestic public financing predominates. Further, no pooled or coordinated global financing mechanism currently exists for NTD medicines, leaving supply fragmented and reliant on donations. Existing donation programs for albendazole, mebendazole, and ivermectin have achieved wide-scale coverage and impact but have not fostered sustainable domestic procurement or integration into primary health care systems.

**Conclusions:**

Introduction of the FDC in the absence of donation schemes poses significant financing and supply challenges, especially for LICs and LMICs where essential medicine availability is already limited. However, it also presents an opportunity to reset NTD access models by embedding sustainable financing strategies, such as pooled procurement, tiered pricing, and primary health care integration, from the outset. Early planning with manufacturers, donors, and governments will be essential to ensure equitable access and country ownership of this novel treatment.

## Author summary

Medicine donation programs have been essential for controlling neglected tropical diseases by enabling mass treatment in many low- and middle-income countries. They can also mask weaknesses in sustainable access to and utilization of medicines.

In this study, we reviewed the current donation landscape for albendazole, mebendazole, and ivermectin, medicines used for treating intestinal worms (STH) and other parasites to better understand the implications for introducing a new fixed-dose combination tablet of ivermectin and albendazole, which may not be donated. Using published evidence, policy reports, and global datasets on essential medicine availability and health financing, we show that the countries with the highest STH burden often have the lowest sustained availability of essential medicines such as these. We also found that health systems in these settings rely heavily on out-of-pocket spending and external support, with limited domestic public financing for medicines, making routine purchasing of a new treatment difficult.

Our findings suggest that planning for the availability of a new STH medicine must be done with careful consideration of the current and evolving donation and financing contexts, creating a timely opportunity to build more sustainable access models from the outset.

## Introduction

Donated medicines have underpinned efforts to prevent and treat neglected tropical diseases (NTDs) in low- and middle-income developing countries [1–6]. Over recent decades, pharmaceutical companies have donated billions of treatments for diseases like soil-transmitted helminths (STH), onchocerciasis (oncho), and lymphatic filariasis (LF), while significantly advancing global health goals [1,7,8]. The pharmaceutical industry’s commitment to NTDs started when Merck decided in 1987 to donate ivermectin, under the trade name Mectizan [9]. Other donation commitments followed and were amplified by the London Declaration in 2012, which led to donations or expanded commitments for 17 different medicines by 13 companies linked to WHO-established NTD goals [1,7].

Part of the benefit of donation programs is their compelling return on investment demonstrated through improved public health outcomes, enhanced corporate social responsibility profiles, and strengthened partnerships with global health organizations [1]. A dollar spent by governments or foundations towards operational costs can leverages medicines potentially valued at over $20 [1,2,10]. For pharmaceutical partners, these programs can also indirectly support brand reputation, stimulate demand for complementary products, and align with long-term business strategies by fostering goodwill and trust among stakeholders.

The inclusion of certain NTD treatments in WHO’s Essential Medicines List (EML) highlights the importance of long-term access beyond donation programs [11]. Some developing countries procure NTD medicines independently, in addition to, or in replacement for donation programs such as where there is local production or established national insurance schemes. Some also procure for ages or groups excluded from donation commitments [12]. However, domestic financing remains a critical challenge, particularly for low-income and lower-middle income countries (LICs and LMICs), where external aid and especially out-of-pocket spending may still play disproportionately large roles in health funding [13].

Approximately 1.5 billion people around the world are infected with STH, increasing anemia and impacting growth, development and learning in children. The five main species of STH are *Ascaris lumbricoides, Trichuris trichiura, Strongyloides stercoralis* and the two species of hookworms (*Ancylostoma duodenale and Necator americanus).* These species have been mainly treated in LICs and LMICs with donated medicines such as albendazole or mebendazole. In some countries STH treatment is a byproduct from treatment of other diseases (e.g., LF treatment with albendazole co-administered with ivermectin; onchocerciasis treatment with ivermectin). The current use of monotherapy albendazole or mebendazole for deworming may increase the risk of resistance developing by sub-optimally treating *T trichiura and S stercoralis*, and potentially hookworms which are well treated if ivermectin and albendazole are co-administered [14].

It is within a context of shifting global health resources, calls for alternative access models, and growing concern about suboptimal treatment efficacy [15–21] that the development of a fixed-dose combination of ivermectin and albendazole (FDC) represents both a scientific milestone and a critical test of whether the global health community can move beyond reliance on donations towards a more sustainable and equitable model of medicine access.

### Towards the first new STH treatment in 40 years

Over a decade of work and $15 million have been invested by governments, researchers, and a pharma partner, the Chemo Group, to develop FDC. This new, mango-flavored, orodispersible tablet simplifies logistics and treatment. It removes the need to measure height or weight and adjust the number of pills administered accordingly [19]. The FDC is a single pill of ivermectin/albendazole 9 mg/400 mg for treating people 15 kg to <45 kg and ivermectin/albendazole 18 mg/400 mg tablets for treating people ≥45 kg [20,21]. The FDC is safe and delivers significantly greater efficacy compared to albendazole alone, particularly against *Trichuris trichiura* and *Strongyloides stercoralis* infections [22].

The European Medicines Agency (EMA), working in coordination with WHO through the EU-Medicines for all (EU-M4all) procedure has provided a positive scientific opinion assuring the quality of the medicine for treating STH and LF, and the product is on the path to consideration for WHO prequalification [23]. Ghana licensed FDC in late 2025 and market authorization has and will be sought from regulators in other developing countries, such as Kenya and the Americas.

The EMA subsequent to its positive scientific opinion noted that FDC “*will help reduce the risk of incorrect dosage, improve adherence, and reduce manufacturing and transport costs. Ultimately, this will allow more people to be treated*” (paragraph 7, [23]).

The medicine may not be donated but instead supplied at a quantity and cost, yet to be fully defined, that is sustainable for the manufacturer and aligned to its public health commitments to public sector co-investors in its development. This paper examines the availability of essential medicines in health facilities, prevailing health financing patterns, and the nature of existing NTD donation models, in order to draw implications for the future supply and financing of the newly developed FDC for STH.

## Methods

### Literature search and selection

Relevant literature was identified through structured searches in public databases (PubMed, Google Scholar) and gray literature sources (e.g., WHO, World Bank, global health partner websites) using key terms such as “neglected tropical diseases”, “soil-transmitted helminths” “drug donation programs”, “essential medicines availability”, “health financing”, “low-income countries”, “NTD medicine supply”, “out-of-pocket health spending”, and “global health financing mechanisms” among other terms. Websites were reviewed for qualitative insights on donation programs, and access models. The search strategy combined terms related to STH, medicine donations, sustainable financing, procurement, and essential medicines. Additional materials were identified through snowballing from reference lists and expert consultation. Sources were included if they contributed empirical data, policy analysis, or programmatic insight relevant to STH supply, financing, or medicine access.

### Data sources

Two primary global datasets were used to complement a review of published and gray literature. WHO’s SARA data was extracted, indicating proportions of health facilities reporting sustainable availability of a core set of WHO-recommend essential medicines, which includes those for treating STH [24]. Data were available for multiple countries across WHO regions and categorized by World Bank income groups for comparison and analysis. To ensure relevance for STH, we cross-checked the dataset against WHO reports [25] and published literature [26–28] to exclude countries without reported STH preventive chemotherapy (PCT) in the WHO PCT databank or WHO-regional-level studies with country-specific prevalence analyses for STH. Based on these criteria, two countries, Lebanon and Mongolia, were removed from the data.

We also extracted national-level health expenditure data, disaggregated by financing source (domestic public, domestic private/out-of-pocket, and external aid) and categorized by country income groups (2024 classification) from the World Bank’s World Development Indicators [13,29]. These data informed analysis of health spending patterns relevant to potential FDC uptake.

A review of 35 sources, including peer-reviewed papers, gray literature, and institutional websites literature searches provided the primary data for analysis of existing donation programs and their implication for FDC.

### Data processing and analysis

Analyses were carried out using Microsoft Excel LTSC MSO (Version 2508). For SARA data we used each country’s most recent survey where multiple surveys existed. Country income categories adhered to World Bank’s categories of low- (LIC), lower-middle (LMIC), upper-middle (UMIC), and high-income countries (HIC).

Findings were synthesized using three variables shaping sustainable access to STH medicines: Facility-level medicine availability, health financing mechanisms across World Bank income levels, and donation models from existing STH treatment programs. This framework guided integration of quantitative data (SARA, World Bank) with qualitative insights from the literature review, ensuring a comprehensive analysis of implications for future FDC supply and financing.

## Results

Analyses of current STH supply, financing, and donations suggest three primary groups of findings that will inform FDC. These are essential to consider, but not exhaustive. They consider availability and funding of supplies for other WHO-defined essential medicines, health spending patterns in countries where FDC might be implemented, and the potential for countries to continue relying on donations of monotherapies for deworming.

### Availability in health facilities and funding for other WHO-defined “essential medicines”

Analyses of essential medicine, including albendazole and ivermectin, availability and affordability from the WHO SARA [24] assessments, together with aggregate estimates of national spending on health care, provide important insights for understanding the feasibility of implementing FDC, particularly through primary health care systems.

The SARA data, presented in Table 1 below, revealed substantial variation in the availability of essential medicines in health facilities across World Bank income groups. In LICs such as Burundi, Burkina Faso, and Mali, the proportion of facilities reporting sustained availability of core essential medicines was consistently 0% during the assessed periods. Similarly, in several LMICs essential medicine availability and affordability may not be sustained. Countries such as Haiti and Tanzania reported zero availability while Bolivia and Lao reported 23% and 25% availability.

**Table 1:** Proportion of health facilities with a core set of relevant essential medicines available and affordable on a sustainable basis.

Country/Location	WB Income Group ^a^	WHO Region	Year of survey ^b^	Proportion
Burundi	LIC	Africa	2016	0.0
Burkina Faso	LIC	Africa	2016	0.0
Mali	LIC	Africa	2016	0.0
Congo (Brazzaville)	LMIC	Africa	2016	0.0
Ghana	LMIC	Africa	2016	12.5
Guinea	LMIC	Africa	2016	12.5
Senegal	LMIC	Africa	2016	7.7
Tanzania	LMIC	Africa	2016	0.0
Zambia	LMIC	Africa	2016	16.7
Mauritius	UMIC	Africa	2008	26.2
Bolivia	LMIC	Americas	2016	23.1
Colombia	UMIC	Americas	2016	8.3
Ecuador	UMIC	Americas	2016	50.0
Haiti	LMIC	Americas	2011	0.0
Peru	UMIC	Americas	2016	69.2
Chile	HIC	Americas	2016	36.4
Trinidad and Tobago	HIC	Americas	2016	0.0
Sudan	LIC	Eastern Mediterranean	2013	34.5
Kyrgyzstan	LMIC	Europe	2016	0.0
Tajikistan	LMIC	Europe	2013	15.0
Moldova	UMIC	Europe	2019	21.7
Indonesia	UMIC	South-East Asia	2010	14.7
Lao	LMIC	Western Pacific	2013	25.3

^**a**^ World Bank country income level classification 2024 [29]; ^b^ Latest year SARA survey.

These data are consistent with analyses that public healthcare spending at 11.2% globally in 2021, has been well below targets of 15% of national budgets in every region of the world [30]. In Africa, countries spend only 7.3% of budgets on public healthcare with many well below that, 8.1% in South-East Asia, 10% in the Eastern Mediterranean, 10.2% in the Western Pacific, and 14.7% in the Americas.

The evidence spanning many years suggests the foundational infrastructure for sustaining access to essential medicines is weakest in the very geographies where the burden of STH is highest. This presents a structural paradox for the introduction of a nondonated FDC. While it is programmatically superior and clinically more effective than monotherapy, it will be competing for space in fragile procurement systems that already fail to deliver established essential medicines and public healthcare services.

### Health spending patterns in countries where FDC might be used

**Fig 1:**
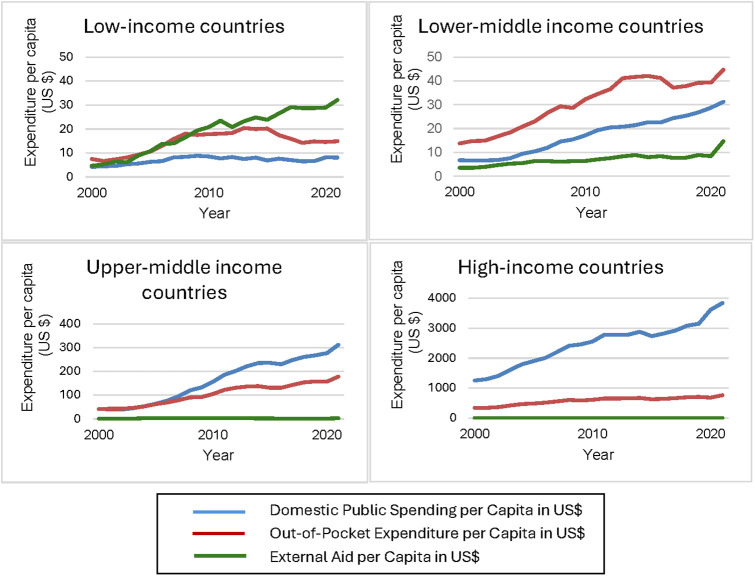
Health expenditure by World Bank income groups.

Spending patterns across country income groups highlight the varying financing gap that will be exacerbated for an STH medicine to be purchased by countries (Fig 1). Whereas high (GNI/Capita > $13,935) and upper-middle income countries (GNI/Capita between $4,496 and $13,935) rely to a large extent on domestic public spending, low-income countries (GNI/capita ≤ $1,135) depend largely on external aid, including donated medicines, and out-of-pocket expenditure. Lower-middle income countries (GNI/capita between $1,136 and $4,495), on the other hand, have seen growth in both out-of-pocket expenditures and domestic public spending, yet out-of-pocket remain 50% higher than domestic spending and many-fold higher than external support [31]. These trends highlight the challenge of shifting away from donor-reliant models for NTDs, such as to out-of-pocket payments in LICs and LMICs [32], especially when compared to the well-documented progress on countries co-financing vaccines with Gavi or aspirations of centralized procurement to improve efficiencies [33,34].

In-country funding patterns show that out-of-pocket expenditure far exceeds domestic public funding in LICs and LMICs. Individuals choose to spend scarce funds on curative treatments (e.g., when sickness impacts daily life). As many people will not realize they or their child has an STH infection, it is unlikely families and communities will prioritize treatment for out-of-pocket spending in LICs and LMICs. This suggests that FDC implementation cannot only rely on current domestic health funding patterns, at least in the countries where it is most needed.

Although Gavi has demonstrated that LMICs can progressively take on greater responsibility for vaccine procurement [35], a similar model has not been developed for NTD medicines. There has also been no global commitment over recent decades to a joint, centralized financing and procurement mechanism for NTD medicines, like Gavi, the Global Fund or the Stop TB Partnership’s Global Drug Facility, nor a revolving procurement fund as the Pan-American Health Organization uses to support reliable vaccine procurement [36]. Taken together, it leaves NTD medicine supply and financing relatively fragmented and historically exacerbated the reliance on donation models.

### Potential to continue monotherapy through medicine donations

A FDC of ivermectin and albendazole will need to be considered in light of existing, complementary donation programs and their potential directions in the future. Iterative discussions will be necessary to determine how best to achieve shared public health goals through available medicines in each country. Decisions on donation quantities to each country are generally mediated or coordinated through WHO, but are reliant on available epidemiological data that may be years old.

#### Albendazole.

GSK committed up to 200m tablets per year to 2025 for STH in school-age children and preschool-age children, and up to 100m per year from 2026-2030, with actual amounts provided according to country demand. It has donated over 12 billion albendazole tablets for LF, targeting adults and children. GSK is committed to donating albendazole until LF is eliminated as a public health problem *(Personal communication – Minne Iwamoto, GSK)*. Treatments have gone to more than 50 countries [37].

#### Mebendazole (Vermox).

Mebendazole donation program targets children, especially those of preschool and school age. Johnson & Johnson (J&J) has donated over 2.4 billion doses of mebendazole to more than 60 countries to fight STH. A key commitment is also the current transition to a chewable mebendazole tablet, which simplifies administration to children as young as one year and improves treatment adherence. J&J plans to donate up to 200 million doses annually through 2030, aiming for one billion more doses. This effort supports the WHO’s 2030 NTDs roadmap [38].

#### Mectizan (Ivermectin) donation program.

Since 1987, Merck has committed to donating ivermectin indefinitely to combat onchocerciasis (river blindness) [39]. In 1998, the company expanded its commitment to include LF by co-administering IVM with albendazole (e.g., donated by GSK) in African countries and Yemen where the disease is co-endemic with onchocerciasis [9]. From 2017 to 2025 and further extended through 2030, Merck pledged up to 100 million treatments for “triple therapy” (IVM, diethylcarbamazine, and albendazole), following new WHO recommendations for the acceleration of LF elimination in countries where onchocerciasis is not endemic.

The Mectizan Donation Program (MDP) now reaches over 300 million people annually [9], with over 14 billion ivermectin tablets shipped to 62 countries and territories since its inception *(Personal communication – Yao Sodahlon, MDP)*. These efforts have contributed to Colombia, Ecuador, Guatemala, Mexico, and Niger successfully eliminating river blindness and stopping treatment, while Senegal is in post-treatment surveillance due to the partnership. Seven other countries have stopped treatment in some areas, as treatment has led oncho to become more focal [40].

Despite this progress, much remains to be done. In 2024, Merck partnered with 36 countries and territories globally, approving 406 million treatments for river blindness and LF, of which 274 million are IVM for oncho, 79 million are for IVM and ALB for only LF and/or places co-endemic with oncho and LF, and 53 million are for triple therapy [40–42].

#### Targeting by donation programs.

The WHO EML states that albendazole and ivermectin can be used together to improve efficacy of STH treatment and reduce the risk of resistance [11]. However, there is no donation program that provides both for STH. Deworming programs only donate albendazole or mebendazole. Ivermectin is donated only for treatment of onchocerciasis or in coordination with albendazole for treatment of LF. Many populations are treated for STH as a secondary effect where onchocerciasis and/or LF is endemic. STH donation programs also generally only cover parts of populations that ideally would be treated (e.g., treating children, but not women of reproductive age).

## Discussion

The data above suggest that current financing and supply models for NTD medicines are unlikely to lead to wide access to FDC and the benefits noted by the EMA when providing its scientific opinion [23]. The challenges for FDC are not unique to it, yet early and intentional planning could lend the global health community an opportunity to accelerate the re-engineering of access pathways for NTD medicines, contributing to a shift from reliance on donation-driven access to models that embed country ownership, sustainability, integration, and country-specific approaches based on local data from the outset.

The findings suggest integration in national systems, such as through EMLs, will not address the gaps in the countries where FDC is most in need. Integration may increase national ownership if complemented by financing and supply mechanisms that remove or subsidize purchase costs for some countries, and pooled procurement models that may help lower prices and increase demand predictability [43,44]. More current, country-specific and focal estimates of disease burden, especially for STH, even through modeling, and integration into national health information systems might increase ownership since current gaps in data may further undermine governments’ ability to prioritize and budget for new medicines.

Fully exploring financing options for FDC requires dedicated research and publication, but current health financing shapes the prospects for FDC adoption. In most LICs and LMICs, medicine payments are drawn from a complex mix of government and nongovernment funds, but with out-of-pocket spending by households already carrying the largest burden due to constrained fiscal space and competing health priorities [45–48]. Wealthier countries (UMICs and HICs) generally spend more per capita on essential medicines but benefit from lower relative prices due to purchasing power and stronger procurement systems [46,47]. Ironically, poorer countries may pay higher prices proportionally, facing greater affordability challenges [48]. For FDC, these financing realities imply that equitable use in high-burden, resource-limited settings will depend on designing strategies also premised on minimizing household expenditure.

Supply and financing are also informed by WHO recommendations on delivery strategies. It advises that countries consider mass drug administration (MDA) of albendazole or mebendazole where there is medium to high STH prevalence of 20–50%, or a clinic-based approach to delivery in areas with low baseline prevalence (<20%) [49–51]. MDAs end up treating many people who do not have STH and require securing funding for operational costs that can be US$1, or even more per person [52]. For FDC, since the MDA strategy largely depends on donated medicines, its validity and the epidemiological thresholds that trigger deworming interventions may need to be reevaluated in terms of cost-efficiency if drugs are not donated. Consequently, delivery in low-prevalence areas is premised upon effectiveness and reach of existing health infrastructure. Primary health care integration of STH has not been extensively modeled or costed, but may provide a more cost-efficient means for targeting the most at-risk populations in communities, such as treating any young children already coming for preventative or curative services, and women of reproductive age coming to clinics, such as when bringing children or seeking birth control medicines.

The STH and LF medicine donation programs have expanded access effectively, but they do not provide a clear pathway to national financing or sufficient incentives for investing in data systems that can demonstrate when medicines are no longer needed [53]. In contrast, a nondonation model for a product like FDC that offers programmatic efficiencies and broader efficacy [22], may offer an opportunity to reset relationships by embedding sustainability plans and stronger targeting of medicines, integration, and country ownership from the start.

### Study limitations

The structured approach and triangulation across multiple data sources strengthen the validity of findings. Nonetheless, the analysis should be interpreted in light of the modest but very diverse cross-section of countries in the SARA dataset spanning a decade, with some countries lacking recent data and substantial variation in survey years, which may limit further comparability. It is worth noting that we attempted using data from the Demographic and Health Survey’s Service Provision Assessment (SPA), but it lacked specific data on essential medicine availability in facilities. We also reviewed individually published studies reported in the literature. They were limited in coverage, such as focusing on a narrow sub-national areas, [54,55], also used the SARA dataset [56] or were not clearly inclusive of STH-related medicines [55,57].

## Conclusion

Without a donation mechanism, access where most needed will likely depend on the extent to which the use of FDC is owned by health systems, and supported with a tailored mix of strategies according to the contexts of different groups of countries. Fully analyzing such a mix is beyond the scope of this paper. However, the underlying analyses presented here map the scale of challenges and hint at opportunities ahead for FDC supply and financing. It provides a baseline upon which tailored approaches can be built, informing novel supply and financing strategies that could be pursued in coming years for FDC and other NTD-related innovations in diagnostics and medicines. As an era of reliance on large centrally organized global funds transitions to a new norm, such tailored approaches may become ever more prevalent and critical for ensuring access to novel global health interventions.

**Key learning points**

**1. Essential medicine availability remains weakest where STH burden is highest**

The study showed that low- and many lower-middle income countries, which carry the greatest STH burden, consistently report investments well below targets for public health programs and the lowest sustainable availability of essential medicines. This structural weakness poses a direct challenge for introducing a nondonated FDC, underscoring the need to pair its roll-out with health system strengthening, reliable procurement pathways, and national-level prioritization.

**2. Financing models are fragmented and inequitable**

Essential medicine financing in endemic countries relies heavily on out-of-pocket spending, with limited public budgets and insurance or pooled procurement schemes. This creates financial barriers to sustained access and risks excluding the most vulnerable populations. For FDC to be viable, financing strategies must explicitly reduce household costs through public investment, pooled procurement, and innovative prepayment mechanisms.

**3. FDC offers an opportunity to re-engineer access pathways for NTD medicines**

By not being donation-based, the FDC can help contribute to shifts away from externally reliant models that may have historically delayed country ownership and system integration. Its introduction presents a critical moment to embed sustainability from the outset and accelerate progress on equitable access to future NTD innovations.

**Five key papers in this field**

1 Bradley M, Taylor R, Jacobson J, Guex M, Hopkins A, Jensen J, *et al*. Medicine donation programmes supporting the global drive to end the burden of neglected tropical diseases. *Trans R Soc Trop Med Hyg*. 2021;115(2):136–44. https://doi.org/10.1093/trstmh/traa167
2 Sodahlon Y, Ross DA, O’Carroll P, McPhillips-Tangum C, Lawrence J, Tucker A, *et al*. Sustaining the gains achieved by national neglected tropical disease (NTD) programs: how can we build NTD program country ownership and sustainability? *PLoS Negl Trop Dis*. 2024;18(6):e0012211. https://doi.org/10.1371/journal.pntd.0012211
3 World Bank. Paying for essential medicines for PHC. Washington DC, USA: World Bank; 2022. Available from: https://thedocs.worldbank.org/en/doc/7694bee9048fcc014ad3157d59e1f9fc-0200022022/related/AHFF-PS4-Background-Note-Final.pdf
4 Human Rights Watch. Global failures on healthcare funding | Human Rights Watch [Internet]. 2024 [cited 2026 Jan 14]. Available from: https://www.hrw.org/news/2024/04/11/global-failures-healthcare-funding
5 Lin WM, Addiss DG. Sustainable access to deworming drugs in a changing landscape. *Lancet Infect Dis*. 2018;18:e395–8. https://doi.org/10.1016/S1473-3099(18)30351-7
